# Intelligent Corrosion Sensing and Detection for Aerospace Ground Equipment: A YOLOv11-Based Framework with Shallow Attention and Bidirectional Feature Fusion

**DOI:** 10.3390/s26175504

**Published:** 2026-08-30

**Authors:** Fang Fang, Dongping Sun, Mingyang Geng, Zhaoyang Qu, Shanzhi Gu

**Affiliations:** 1 School of Chemistry and Chemical Engineering, Nanjing University of Science and Technology, Nanjing 210094, China; sundpe301@163.com; 2 Xichang Satellite Launch Center, Xichang 615000, China; 3 College of Computer Science and Technology, National University of Defense Technology, Changsha 410073, China; qzy@nudt.edu.cn (Z.Q.); gushanzhi17@nudt.edu.cn (S.G.)

**Keywords:** aerospace corrosion detection, irregular defect localization, micro-target detection, diffusion-based augmentation, saliency-based attention

## Abstract

Aerospace ground facilities operating in harsh "three-high" marine environments face rapid corrosion that threatens structural safety. Automated visual inspection is fundamentally hindered by two intertwined challenges: extreme scarcity of annotated real samples, and the intrinsic complexity of corrosion targets themselves—micro-pitting and fine cracks occupy only a handful of pixels with signals easily overwhelmed by complex backgrounds, while mature corrosion regions exhibit extreme geometric irregularities that defeat conventional detection and regression methods. To tackle these challenges, this paper proposes CorrSense, a YOLOv11-based framework with three synergistic innovations. First, we establish a dual-path data ecosystem combining real acquisition with diffusion-driven augmentation that synthesizes visually realistic and structurally consistent corrosion samples to expand training distribution. Second, we develop a saliency-based feature enhancement strategy using parameter-free SimAM attention in shallow layers, which amplifies micro-corrosion responses while suppressing background activations with zero parameter overhead. Third, we formulate a dual-pronged geometric calibration strategy: a customized BiFPN with learnable weighted fusion for dynamic cross-scale feature orchestration, coupled with a morphology-adaptive CIoU loss that modulates aspect ratio constraints according to target shape. Extensive experiments demonstrate that CorrSense consistently outperforms state-of-the-art detectors including YOLOv8, YOLOv9, and RT-DETR on challenging samples, particularly on irregularly shaped corrosion and micro-pitting targets where competing methods typically struggle, validating its effectiveness as a promising solution for intelligent corrosion inspection in coastal launch sites.

## 1. Introduction

Aerospace ground facilities, including launch towers, umbilical arms, propellant loading systems, and associated service structures, constitute the critical infrastructure that underpins the success of contemporary space missions. As the frequency and complexity of space exploration continue to escalate, the health monitoring of these facilities has become increasingly vital to mission assurance and operational safety. However, these structures are chronically exposed to extreme marine atmospheric conditions, particularly at coastal launch sites such as the Wenchang Space Launch Site in China, where high temperature, high humidity, and high salt spray concentration, collectively referred to as the “three-high” environment, synergistically accelerate electrochemical corrosion on metallic structural components. This corrosion manifests in various forms, ranging from localized pitting and stress corrosion cracking to generalized diffuse corrosion, all of which progressively degrade material integrity, compromise load-bearing capacity, and ultimately pose serious threats to the safety, reliability, and service life of launch facilities. Traditional manual inspection methods, heavily reliant on visual examination by trained personnel or conventional non-destructive testing techniques, are increasingly inadequate given the scale and inaccessibility of modern launch complexes, resulting in excessive time consumption, high labor costs, and inherent subjectivity that often leads to inconsistent and potentially unreliable assessments.

Substantial research efforts have been devoted to automated surface defect detection using deep learning. The rapid advancement of deep learning technologies in recent years [1,2,3,4] has fundamentally transformed the landscape of computer vision, enabling unprecedented performance in tasks ranging from image classification and object detection to semantic segmentation. Generic object detection architectures such as YOLO [5], Faster R-CNN [6], and RT-DETR [7] have been widely adopted as backbones for defect detection, often combined with attention mechanisms or feature pyramid networks to enhance multi-scale perception. However, the direct transfer of these methods to aerospace corrosion detection is problematic for several reasons. Most existing industrial defect datasets predominantly feature well-defined defects on relatively uniform backgrounds with controlled illumination, whereas aerospace corrosion inspection must contend with highly irregular defect morphologies, extreme scale variations, and severe background clutter. Furthermore, the vast majority of current approaches assume the availability of large-scale, densely annotated training data, an assumption that fails dramatically in the aerospace domain, where corrosion samples are scarce, acquisition is constrained by launch schedules and safety protocols, and annotation requires domain-expert knowledge of corrosion mechanisms and progression patterns.

**Figure 1:**
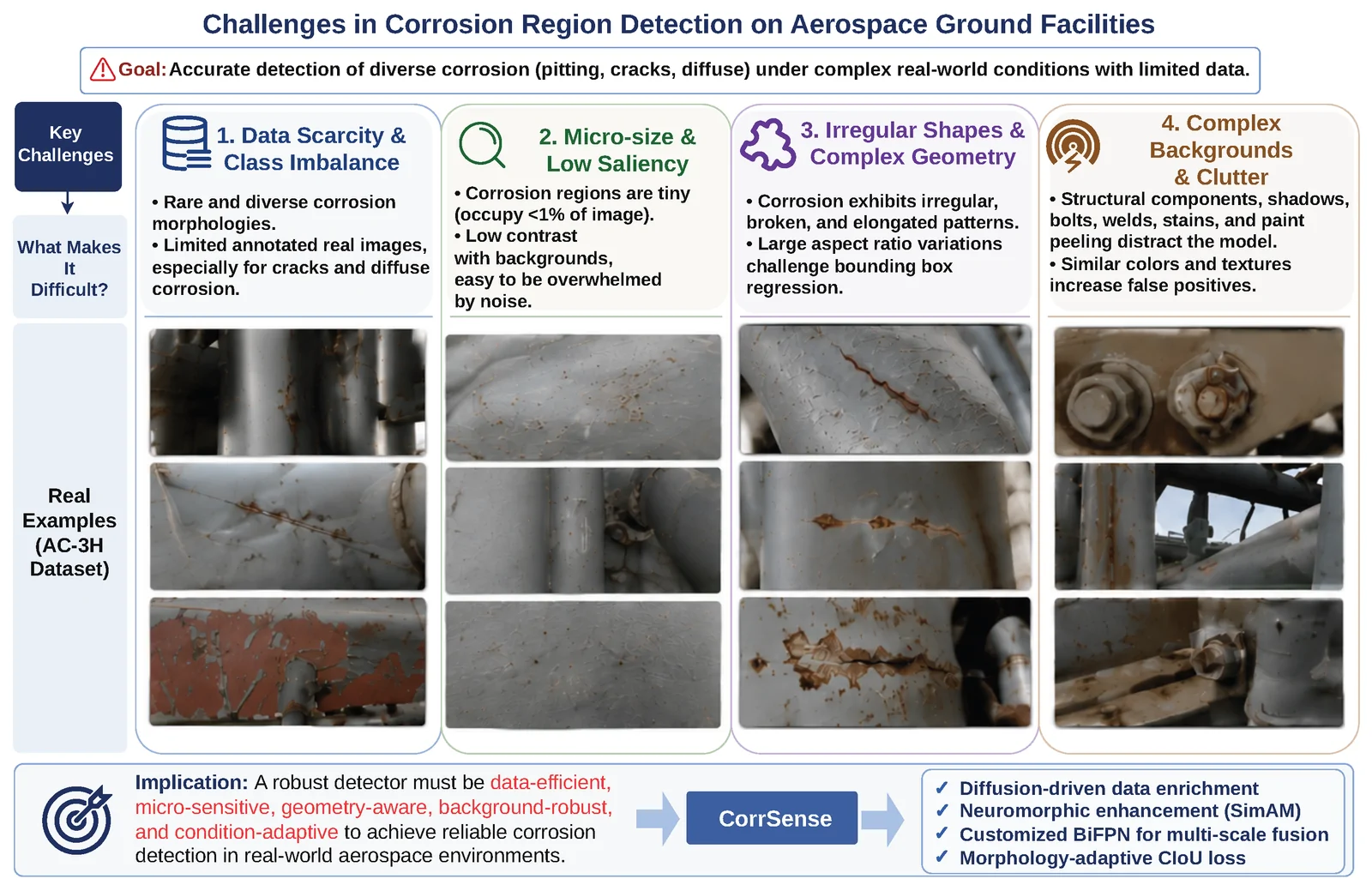
Illustration of our motivation.

As shown in Figure 1, the unique challenges of aerospace corrosion detection extend far beyond the general difficulties of industrial defect inspection. First, early-stage corrosion predominantly manifests as micro-pits and fine cracks that occupy merely a handful of pixels in standard-resolution images, whose exceptionally weak signals are easily overwhelmed by complex backgrounds such as machining textures, weld seams, bolt edges, and specular reflections induced by the highly reflective metallic surfaces of launch structures. Second, mature corrosion regions exhibit extreme geometric irregularity: elongated stress corrosion cracks present aspect ratios far exceeding typical values with narrow, thread-like morphologies that challenge conventional receptive fields designed for compact objects; diffuse corrosion areas display ambiguous, gradually transitioning boundaries rather than well-defined contours, making precise bounding box delineation inherently difficult. These characteristics cause conventional bounding box regression losses to fail in providing effective geometric constraints, leading to significant localization errors. Third, the severe scarcity of annotated real corrosion samples, due to high acquisition costs, restricted access to active launch sites, and the logistical difficulties of capturing rare corrosion morphologies under operational conditions, makes it exceedingly difficult to train robust deep learning models with sufficient generalization capacity. These three challenges, namely data scarcity, micro-target perceptual saliency, and irregular morphology localization, collectively form a set of intertwined bottlenecks that require a holistic, systematically designed solution.

To comprehensively address these challenges, this paper proposes CorrSense, a YOLOv11-based intelligent corrosion sensing and detection framework that integrates three synergistic innovations. First, we construct a dual-path data ecosystem combining real-world acquisition with diffusion-driven generative augmentation [8], establishing AC-3H, the first dedicated aerospace corrosion dataset for “three-high” environments, and coupling LoRA-adapted Stable Diffusion with DDIM inversion to synthesize physically plausible and structurally consistent corrosion samples that expand training distribution to cover rare morphologies and extreme cases. Second, we develop a saliency-based feature enhancement strategy using parameter-free SimAM attention embedded in shallow backbone layers, which amplifies neuronal responses at locations deviating from background statistics, precisely where micro-corrosion resides, while suppressing uniform background activations with zero parameter overhead. Third, we formulate a dual-pronged geometric calibration strategy: replacing the native PANet with a customized BiFPN that prunes the coarse C5 layer and introduces learnable weighted fusion for dynamic cross-scale feature orchestration, coupled with a morphology-adaptive CIoU loss that modulates aspect ratio constraints according to target shape, intensified for elongated cracks, relaxed for compact pitting, and balanced for diffuse corrosion. Extensive experiments on the AC-3H test set demonstrate that CorrSense consistently outperforms state-of-the-art detectors, including YOLOv8, YOLOv9 [9], RT-DETR [7], and the baseline YOLOv11 [10], achieving substantial improvements in both detection accuracy and localization precision. Notably, on challenging samples characterized by extreme aspect ratios or ambiguous boundaries, our method achieves particularly significant gains over competing approaches. These results collectively validate that the proposed framework shows promise in addressing the intertwined challenges of data scarcity, micro-target saliency, and irregular morphology localization, providing a promising approach with potential for deployment in coastal launch sites, with potential transferability to other industrial inspection scenarios characterized by similar difficulties.

The contributions of this paper are threefold.

- **A dual-path data ecosystem with diffusion-driven augmentation.** We construct AC-3H, the first dedicated corrosion dataset for aerospace facilities under “three-high” environments, and design a diffusion-driven augmentation engine coupling LoRA-adapted Stable Diffusion with DDIM inversion to synthesize physically plausible corrosion samples. This effectively addresses data scarcity and expands training distribution to cover rare corrosion morphologies, substantially improving model generalization on challenging samples.
- **A saliency-based feature enhancement strategy with parameter-free SimAM.** We embed SimAM attention into shallow backbone layers to selectively amplify micro-corrosion responses at statistical outliers while suppressing background activations. This zero-parameter mechanism enhances small-target recall without increasing computational overhead, consistently outperforming conventional parameterized attention modules.
- **A dual-pronged geometric calibration strategy with customized BiFPN and morphology-adaptive CIoU.** We replace PANet with a customized BiFPN featuring learnable weighted fusion and layer pruning, and propose a morphology-adaptive CIoU loss that modulates aspect ratio constraints according to target shape. This framework significantly improves localization precision for irregularly shaped corrosion targets, achieving substantial gains over state-of-the-art detectors including YOLOv8, YOLOv9, and RT-DETR on challenging samples.

The remainder of this paper is structured as follows. Section 2 reviews the background and related work on aerospace equipment corrosion detection and computer vision methods for surface defect inspection. Section 3 presents the proposed CorrSense framework in detail, including the dual-path data ecosystem with diffusion-driven augmentation, the saliency-based feature enhancement strategy with SimAM, and the dual-pronged geometric calibration approach with customized BiFPN and morphology-adaptive CIoU loss. Section 4 describes the experimental setup, datasets, evaluation metrics, and implementation details, followed by comprehensive results and analysis including ablation studies and comparisons with state-of-the-art methods. Section 5 concludes the paper and discusses future research directions.

## 2. Related Work

### 2.1. Aerospace Corrosion Detection

Corrosion has long been a critical concern for the aerospace industry, as it directly compromises the structural integrity, operational safety, and service life of aircraft and ground support equipment [11,12,13,14,15]. Aerospace structures are frequently exposed to aggressive marine environments characterized by salt spray, humidity, and temperature extremes, which significantly accelerate electrochemical corrosion processes. Traditional inspection methods primarily rely on manual visual examination and conventional non-destructive testing (NDT) techniques, including ultrasonic testing, eddy current, radiographic inspection, and infrared thermography. Notably, visual inspections constitute more than 80% of the overall inspection procedures for large transport-category aircraft, yet they remain inherently subjective, time-consuming, and heavily dependent on inspector experience [16].

In response to these limitations, deep learning has emerged as a transformative solution for automated corrosion assessment due to its ability to extract features, segment semantics, and perform prediction through high-dimensional image data [11,17,18]. Recent reviews have comprehensively summarized progress in deep learning architectures for aircraft corrosion detection, including Convolutional Neural Networks (CNNs) [19], Fully Convolutional Networks (FCNs) [20], U-Net variants [21], Vision Transformers (ViTs) [22], and ensemble models [11]. Key enabling strategies identified include advanced data augmentation, transfer learning, and the construction of corrosion-specific annotated datasets to address data scarcity and environmental variability. However, the review also notes that challenges including data scarcity, environmental variability, and model interpretability persist as major obstacles for safety-critical aerospace applications [11].

Several studies have demonstrated the feasibility of deep learning for aerospace surface defect detection. A comprehensive review by Nascimento et al. explores the utilization of CNN, YOLO, GAN, and other deep learning architectures for detecting defects on aircraft surfaces, covering categories such as dents, scratches, rivet damage, and surface corrosion [16]. [23] provided a systematic review of metal corrosion identification methods on aircraft wing surfaces, analyzing the recognition principles and application scenarios of YOLO, DETR, and other methods, while particularly emphasizing the importance of data augmentation and transfer learning for addressing dataset insufficiency. Most recently, PWDE-YOLOv8n was proposed as an enhanced approach for surface corrosion detection in aircraft cabin sections, incorporating omnidirectional attention mechanisms and improved loss functions to address challenges such as diverse corrosion morphologies, low background contrast, and poor image quality [24,25]. Additionally, [23] proposed a deep learning framework integrating adaptive deblurring with corrosion rating classification, using NAFNet for deblurring and a residual-based classification network with efficient channel attention (ECA), achieving an accuracy of 91.57% in corrosion rating classification.

Despite these advances, several limitations persist. First, most existing methods are evaluated on laboratory-prepared or simulated corrosion samples rather than real-world operational data from active aerospace facilities, limiting their practical applicability. Second, the irregular morphologies of corrosion, characterized by ambiguous boundaries and extreme aspect ratios, are rarely explicitly addressed in existing detection frameworks. Third, there is a notable lack of publicly available, dedicated corrosion datasets for aerospace applications, which constrains model development and comparative evaluation. The AC-3H dataset proposed in this work directly addresses this gap by providing real corrosion images collected from an operational space launch site under “three-high” environmental conditions.

### 2.2. Deep Learning-Based Defect Detection

The field of visual defect detection has been revolutionized by deep learning-based object detection architectures [26]. These algorithms can be broadly divided into two categories: two-stage methods such as Faster R-CNN, which employ region proposal networks for candidate generation followed by separate classification and regression heads, and one-stage methods such as the YOLO (You Only Look Once) family, which directly predict bounding boxes and class probabilities in a single forward pass, offering a favorable balance between detection accuracy and inference speed [27].

Among these, the YOLO family has emerged as a particularly attractive choice for industrial inspection applications due to its superior trade-off between accuracy and efficiency. YOLOv11, the latest advancement in the series, has demonstrated strong performance in various corrosion detection tasks. In the context of reinforced concrete structures, YOLOv11 was successfully applied to analyze 2D B-scan GPR images for automated corrosion detection and severity classification, representing the first computer vision algorithm employed with GPR radargrams for corrosion evaluation. For aircraft applications, PWDE-YOLOv8n was developed with omnidirectional attention mechanisms, achieving substantial improvements over previous YOLO variants in detecting surface corrosion under challenging imaging conditions [23].

Beyond detection architectures, attention mechanisms have been widely adopted to enhance feature representation in defect detection tasks. Squeeze-and-Excitation (SE) networks and Convolutional Block Attention Modules (CBAM) have been integrated into various backbones to improve discriminability [27]. However, these parameterized attention modules introduce additional learnable parameters and computational overhead. Recent work has explored frequency-dilation attention mechanisms that capture both global context and local details while expanding receptive fields [27]. For corrosion rating classification, efficient channel attention (ECA) has been integrated with residual networks to enhance the capture of critical corrosion features, achieving high classification accuracy with reduced computational resources [28].

For multi-scale feature representation, the Feature Pyramid Network (FPN) and its variants, including PANet (Path Aggregation Network), have been widely adopted to enable cross-scale feature interaction. FPN integrates high-resolution and low-resolution features through a top-down path, while PANet incorporates bottom-up augmentation paths to establish bidirectional connectivity. However, these fusion strategies cannot dynamically adjust feature weights according to detection tasks and fail to fully consider the fusion feature context [27].

### 2.3. Data Augmentation and Feature Fusion for Defect Detection

Data scarcity remains a fundamental challenge in defect detection, particularly for safety-critical applications such as aerospace corrosion inspection where real defect samples are rare and acquisition is constrained. To address this, researchers have explored various strategies ranging from traditional augmentation to generative approaches.

Traditional data augmentation techniques such as rotation, translation, and scaling are commonly employed, yet they often lead to overfitting and fail to significantly improve the model’s ability to detect rare or unseen defects [29,30]. More recently, diffusion models have emerged as a powerful alternative for synthetic data generation in industrial defect detection scenarios [31,32]. [16] proposed a diffusion-augmented zero-shot industrial anomaly detection framework integrating similarity-based data screening with a latent diffusion model, achieving state-of-the-art performance on the MVTec AD dataset with a detection speed approximately 8 times faster than the leading zero-shot method. In the context of 3C product surface defects, a diffusion model integrated with similarity search successfully generated high-fidelity synthetic defects, outperforming both classical augmentation techniques and DCGAN-based approaches [26]. These findings suggest that diffusion-based augmentation is particularly effective for handling rare and complex defect types.

For multi-scale feature fusion in defect detection, BiFPN (Bidirectional Feature Pyramid Network) has demonstrated significant advantages over FPN and PANet. BiFPN introduces learnable weighted fusion and bidirectional connections while pruning redundant nodes, achieving more efficient multi-scale feature aggregation with reduced computational cost [33]. In power inspection applications, Bi-YOLOv10 integrated a BiFPN module with a novel Focal-EIoU loss function to address sample imbalance and multi-scale target detection challenges [33]. In UAV aerial image detection, lightweight BiFPN was introduced to enhance multi-scale feature fusion capability, significantly improving small target detection accuracy while reducing model parameters by 34%. [34] developed a lightweight rail surface defect detection algorithm integrating multi-attention mechanisms with content-aware reassembly of features (CARAFE), demonstrating substantial improvements on the S-RSDD dataset while maintaining real-time inference speed of 158 FPS.

Despite these methodological advances, several challenges remain specifically for aerospace corrosion detection. First, the extreme geometric irregularity of corrosion targets, particularly elongated cracks with aspect ratios far exceeding typical values and diffuse corrosion with ambiguous boundaries, poses challenges that conventional bounding box regression losses are ill-equipped to handle. Second, the combination of micro-pitting targets (occupying only a handful of pixels) and large-scale corrosion regions in the same scene demands more flexible multi-scale fusion strategies. Third, the severe scarcity of annotated real corrosion data limits the effectiveness of purely supervised approaches. The CorrSense framework proposed in this work addresses these challenges through a synergistic combination of diffusion-based data augmentation, parameter-free shallow feature enhancement, and morphology-adaptive regression, offering a holistic solution tailored to the unique requirements of aerospace corrosion detection under harsh environmental conditions.

### 2.4. Few-Shot and Knowledge-Driven Defect Recognition

The challenges of limited labeled data and domain shift are common across industrial inspection domains, and recent research has explored various strategies to address these issues. In the context of tool condition monitoring under variable working conditions, the FEK-AML (Fault Evolution Knowledge-driven Adversarial Meta-Learning) method [35] has been proposed for few-shot tool state recognition. This approach explicitly formulates fault evolution knowledge based on the degradation trajectories from healthy states to fault states, and embeds this physical knowledge into a two-stage adversarial meta-learning framework that combines domain adaptation with metric-based meta-learning. The core insight, leveraging knowledge-guided strategies to compensate for limited fault samples, offers methodological inspiration for addressing few-shot and domain-shift problems in other industrial inspection scenarios, including our corrosion detection task.

Drawing inspiration from these principles, our proposed CorrSense framework shares similar motivations in addressing limited data and domain adaptation challenges. The diffusion-driven augmentation can be viewed as a knowledge-guided data generation mechanism, where the LoRA-adapted diffusion model encodes domain-specific corrosion texture priors. While our current work focuses primarily on data augmentation and feature enhancement, the integration of meta-learning and knowledge-driven strategies for cross-site corrosion detection represents a promising direction for future research.

## 3. Methodology

### 3.1. Overall Framework

**Figure 2:**
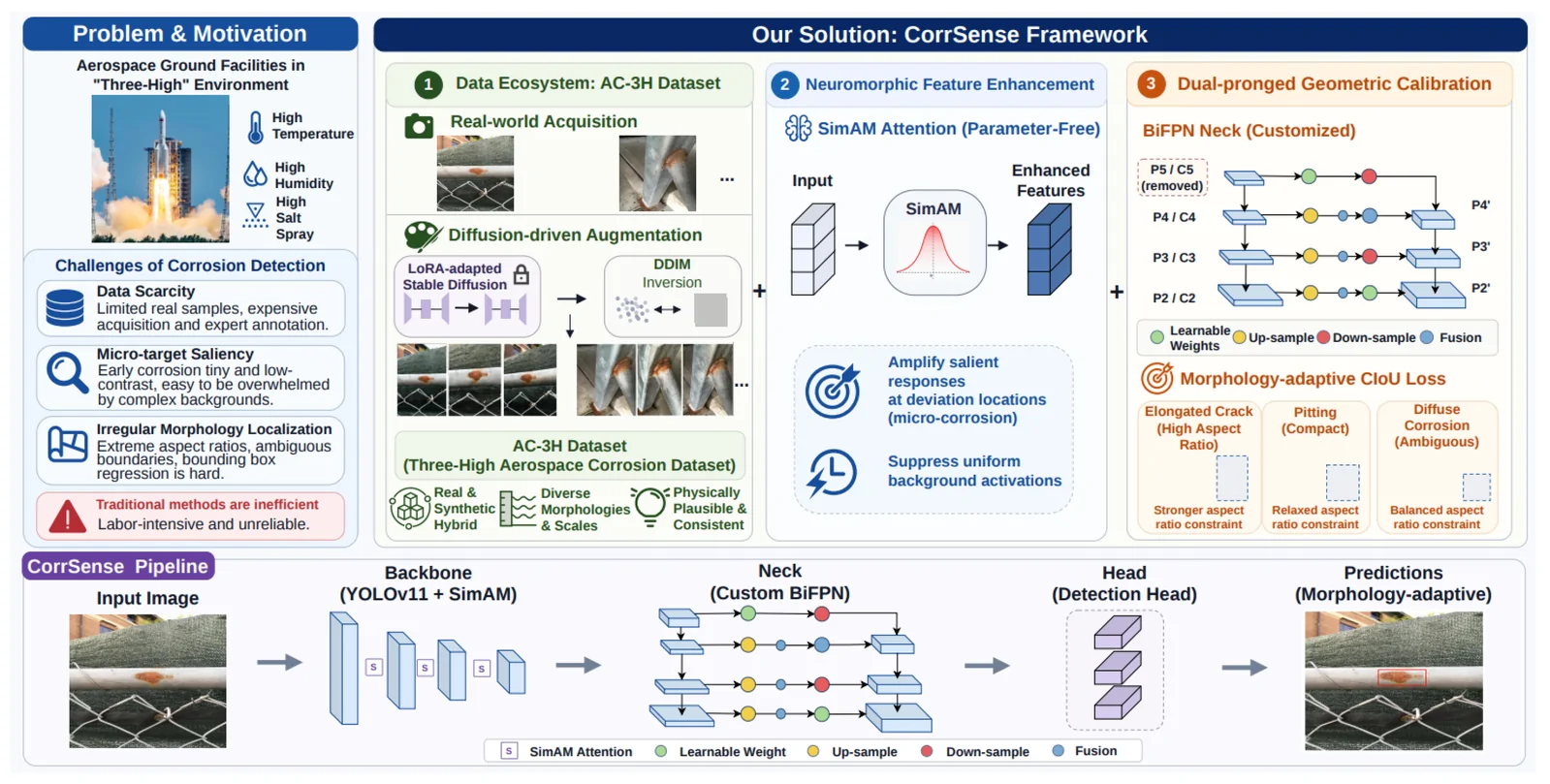
Architecture of CorrSense, which consists of two primary stages: (1) a data preparation and augmentation stage that enriches the training set via diffusion-driven synthesis, and (2) a detection stage that integrates SimAM-enhanced shallow features, a customized BiFPN for dynamic multi-scale fusion, and a morphology-adaptive CIoU loss for precise bounding box regression.

The overall architecture of CorrSense is illustrated in Figure 2. Built upon the YOLOv11 backbone, the proposed framework comprises three cascaded modules that operate sequentially to address the intertwined challenges of data scarcity, micro-target perceptual saliency, and irregular morphology localization. The pipeline begins with a dual-path data ecosystem that integrates real-world acquisition with diffusion-driven generative augmentation, producing a rich and balanced training distribution that covers rare corrosion morphologies. Subsequently, the input image passes through the YOLOv11 backbone, where a saliency-based feature enhancement strategy based on parameter-free SimAM attention is embedded in the shallow C2 and C3 layers to selectively amplify micro-corrosion responses while suppressing background activations. The enhanced multi-scale features are then fed into a customized BiFPN that replaces the native PANet, featuring strategic layer pruning and learnable weighted fusion for dynamic cross-scale feature orchestration. Finally, the detection head produces classification and regression outputs, with the latter supervised by a morphology-adaptive CIoU loss that modulates aspect ratio constraints according to target shape. This end-to-end framework operates with minimal additional computational overhead, as the SimAM module introduces zero learnable parameters, the customized BiFPN maintains efficient feature propagation, and the morphology-adaptive loss only affects training, not inference.

Algorithm 1 summarizes the complete inference and training pipeline of CorrSense.
**Algorithm 1** CorrSense Inference and Training Pipeline  1:**Require:** Input image $I\in R^{3\times H\times W}$, ground-truth boxes $B^{gt}$ (training phase)  2:**Ensure:** Detected corrosion boxes *B* with class scores  3:**Phase 1: Data Augmentation (Offline)**  4:    Generate synthetic samples via diffusion-driven augmentation (Section 3.2)  5:    Filter with FID-based quality control and hybrid annotation verification  6:**Phase 2: Feature Extraction with Shallow Enhancement**  7:    Extract shallow features $C_{2},C_{3}$ from YOLOv11 backbone  8:    Apply SimAM enhancement: ${\tilde{C}}_{2}=\mathrm{SimAM}\left(C_{2}\right),{\tilde{C}}_{3}=\mathrm{SimAM}\left(C_{3}\right)$ (Section 3.3)  9:    Extract deeper features $C_{4},C_{5}$ without enhancement10:**Phase 3: Multi-Scale Feature Fusion**11:    Fuse $\left\{{\tilde{C}}_{2},{\tilde{C}}_{3},C_{4}\right\}$ through customized BiFPN with learnable weights (Section 3.4)12:    Output enhanced multi-scale features $\left\{P_{2},P_{3},P_{4}\right\}$13:**Phase 4: Detection and Regression**14:    Generate predictions {B,cls} via YOLOv11 detection head15:    Compute morphology-adaptive CIoU loss $L_{\mathrm{Corr}-\mathrm{CIoU}}$ (Section 3.4.2)16:    Apply Soft-NMS for final box pruning17:**Return:** Detected boxes *B* with class scores

### 3.2. Dual-Path Data Ecosystem with Diffusion-Driven Augmentation

The severe scarcity of annotated real corrosion samples constitutes a fundamental bottleneck for training robust deep learning models. To overcome this limitation, we establish a dual-path data ecosystem that synergistically integrates real-world acquisition with physics-aware synthetic generation.

On the real-data path, we construct AC-3H (Aerospace Corrosion-3H), the first dedicated corrosion detection dataset for aerospace ground facilities under “three-high” environments. The dataset comprises systematically annotated real images collected from the Wenchang Space Launch Site, covering three representative corrosion morphologies, pitting, cracks, and diffuse corrosion, under diverse illumination conditions and complex structural backgrounds.

On the synthetic-data path, we design a diffusion-driven generative augmentation engine that couples LoRA-adapted Stable Diffusion with DDIM inversion. The core challenge is to generate physically plausible corrosion samples that preserve structural fidelity while expanding the training distribution to cover rare morphologies. Denoising Diffusion Probabilistic Models (DDPMs) define a forward process that gradually adds Gaussian noise to data:

(1) $$q\left(x_{t}|x_{t-1}\right)=N\left(x_{t};\sqrt{1-\beta_{t}}x_{t-1},\beta_{t}I\right),$$

and a reverse process that learns to denoise:

(2) $$p_{\theta }\left(x_{t-1}|x_{t}\right)=N\left(x_{t-1};\mu_{\theta }\left(x_{t},t\right),\Sigma_{\theta }\left(x_{t},t\right)\right).$$

To enable controllable generation, we leverage Stable Diffusion, which performs the diffusion process in a compressed latent space encoded by a pre-trained Variational Autoencoder (VAE), significantly reducing computational cost while preserving high-level semantic structure.

To adapt the pre-trained diffusion model to the aerospace corrosion domain with limited data, we employ Low-Rank Adaptation (LoRA), which constrains the weight update ΔW to a low-rank decomposition:

(3) $$W=W_{0}+\Delta W=W_{0}+BA,$$

where $B\in R^{d\times r}$, $A\in R^{r\times k}$, and the rank r≪min(d,k) significantly reduces the number of trainable parameters while preserving the model’s generative capacity. By fine-tuning only the LoRA parameters on our limited real corrosion samples, the model efficiently captures domain-specific corrosion textures.

To generate synthetic corrosion samples while preserving the geometric structure of pristine facility images, we utilize DDIM (Denoising Diffusion Implicit Model) inversion. DDIM defines a deterministic forward process that maps an input image to a latent noise representation via

(4) $$x_{t+1}=\sqrt{{\bar{\alpha }}_{t+1}}\frac{x_{t}-\sqrt{1-{\bar{\alpha }}_{t}}\epsilon_{\theta }\left(x_{t},t\right)}{\sqrt{{\bar{\alpha }}_{t}}}+\sqrt{1-{\bar{\alpha }}_{t+1}-\sigma_{t}^{2}}\epsilon_{\theta }\left(x_{t},t\right),$$

where ${\bar{\alpha }}_{t}$ are cumulative noise scheduling parameters. The key insight is that this deterministic inversion trajectory encodes the spatial layout of the input pristine image. By starting from this inverted latent code and performing reverse sampling with corrosion-indicating textual prompts as conditions:

(5) $$x_{t-1}={\mathrm{DDIM}}_{\mathrm{reverse}}\left(x_{t},t,{\mathrm{prompt}}_{\mathrm{corr}}\right),$$

the model synthesizes corrosion images that share the same structural layout as the pristine input while exhibiting realistic corrosion textures. This approach ensures that synthetic samples maintain geometric consistency with actual facility structures—crucial for downstream detection tasks.

To guarantee synthetic sample quality, we implement a cascaded quality control mechanism. First, Fréchet Inception Distance (FID) is employed as a distributional filter:

(6) $$\mathrm{FID}=∥\mu_{r}-\mu_{s}{∥}_{2}^{2}+\mathrm{Tr}\left(\Sigma_{r}+\Sigma_{s}-2{\left(\Sigma_{r}\Sigma_{s}\right)}^{1/2}\right),$$

where $\left(\mu_{r},\Sigma_{r}\right)$ and $\left(\mu_{s},\Sigma_{s}\right)$ represent the mean and covariance of real and synthetic feature distributions extracted by an Inception-v3 network. Second, a hybrid annotation verification pipeline combines programmatic initial labeling, lightweight model refinement, and manual inspection to ensure annotation quality. This dual-path ecosystem effectively expands the training distribution to cover rare corrosion patterns, directly addressing the data scarcity challenge.

### 3.3. Saliency-Based Feature Enhancement with SimAM

To amplify the perceptual saliency of micro-corrosion targets against cluttered structural backgrounds, we develop a saliency-based feature enhancement strategy based on the parameter-free SimAM (Simple Parameter-Free Attention Module) mechanism.

The core insight of SimAM is to compute three-dimensional attention weights via an analytical energy function that evaluates the linear separability of each neuron from its surrounding statistical context, a computational motif inspired by neuroscientific principles of spatial inhibition, where neurons that deviate from their local neighborhood are considered more informative. Unlike conventional parameterized attention modules that rely on additional learnable sub-networks, SimAM operates directly on feature statistics, introducing zero learnable parameters.

For an input feature map $F\in R^{C\times H\times W}$, the energy function for each neuron $f_{c}\left(i,j\right)$ is defined as:

(7) $$E_{c}\left(i,j\right)=\frac{{\left(f_{c}\left(i,j\right)-\mu_{c}\right)}^{2}}{\sigma_{c}^{2}+\lambda }+\frac{1}{N},$$

where $\mu_{c}$ and $\sigma_{c}^{2}$ are the mean and variance of the *c*-th channel:

(8) $$\mu_{c}=\frac{1}{H\times W}\sum_{i=1}^{H}\sum_{j=1}^{W}f_{c}\left(i,j\right),\;\sigma_{c}^{2}=\frac{1}{H\times W}\sum_{i=1}^{H}\sum_{j=1}^{W}{\left(f_{c}\left(i,j\right)-\mu_{c}\right)}^{2},$$

N=H×W−1 is the number of spatial positions excluding the target neuron, and λ is a small regularization constant to avoid numerical instability. The first term measures the deviation of the target neuron from the channel mean, normalized by the channel variance; the second term acts as a regularization penalty. A smaller energy value indicates that the neuron is more discriminative relative to its surrounding statistics. Therefore, the attention weight should be inversely related to the energy value.

The attention weight is then computed by inverting the energy such that lower energy values yield higher attention weights:

(9) $$w_{c}\left(i,j\right)=\frac{1}{1+\exp(E_{c}\left(i,j\right))}=\mathrm{sigmoid}\left(-E_{c}\left(i,j\right)\right),$$

followed by channel-wise normalization:

(10) $${\tilde{w}}_{c}\left(i,j\right)=\frac{w_{c}\left(i,j\right)}{\max(w_{c})+\epsilon }.$$

The complete SimAM computation is summarized in Algorithm 2.

By embedding this mechanism into the shallow C2 and C3 layers of the YOLOv11 backbone, we selectively amplify neuronal responses at spatial locations that exhibit statistical deviation from background distributional patterns, precisely where micro-pitting and incipient cracks reside due to their anomalous intensity and gradient characteristics, while simultaneously suppressing homogeneous background activations arising from uniform metal surfaces, repetitive machining marks, and diffuse reflections. Crucially, this saliency bootstrapping is achieved with zero learnable parameters, preserving the model’s architectural compactness and inference efficiency, which is particularly advantageous for resource-constrained edge deployment scenarios.
**Algorithm 2** SimAM Feature Enhancement  1:**Require:** Input feature map $F\in R^{C\times H\times W}$, regularization $\lambda =10^{-4}$  2:**Ensure:** Enhanced feature map $\tilde{F}\in R^{C\times H\times W}$  3:**for** each channel c∈[1,C] **do**  4:      Extract single-channel features $f_{c}\in R^{H\times W}$  5:      Compute $\mu_{c}=\mathrm{mean}\left(f_{c}\right)$ and $\sigma_{c}^{2}=\mathrm{var}\left(f_{c}\right)$  6:      Compute energy:  7:         $E_{c}\left(i,j\right)=\frac{{\left(f_{c}\left(i,j\right)-\mu_{c}\right)}^{2}}{\sigma_{c}^{2}+\lambda }+\frac{1}{H\times W-1}$    8:    Compute attention weights: $w_{c}\left(i,j\right)=\mathrm{sigmoid}\left(-E_{c}\left(i,j\right)\right)$▹ Inverted: lower energy → higher weight    9:    Normalize: $w_{c}=\frac{w_{c}}{\max(w_{c})+\epsilon }$10:      Apply enhancement: ${\tilde{f}}_{c}=f_{c}⊗w_{c}$11:**end for**12:**Return:** Concatenated $\tilde{F}$

### 3.4. Geometric Calibration with Customized BiFPN and Morphology-Adaptive CIoU

To conquer the localization bottleneck imposed by geometric irregularities, we formulate a dual-pronged geometric calibration strategy that addresses the problem from both feature representation and regression supervision perspectives.

#### 3.4.1. Customized BiFPN for Dynamic Multi-Scale Feature Fusion

At the feature representation level, we replace the native PANet with a customized BiFPN that strategically prunes the high-semantic yet spatially coarse C5 layer—whose coarse resolution is ill-suited for localizing fine cracks and micro-pits—and introduces learnable weighted fusion connections, enabling the network to dynamically orchestrate cross-scale feature contributions conditioned on target scale and morphology.

Given input features from three levels $\left\{C_{2},C_{3},C_{4}\right\}$, we construct a weighted BiFPN with explicit intermediate nodes. Following the standard BiFPN formulation, we first remove nodes with only one input edge and add skip connections from the original same-level input to the output node. The top-down pathway produces intermediate features:

(11) $$P_{4}^{td}=\mathrm{Conv}\left(C_{4}\right),\;P_{3}^{td}=\mathrm{Conv}\left(\frac{w_{1}\cdot C_{3}+w_{2}\cdot \mathrm{Upsample}\left(P_{4}^{td}\right)}{w_{1}+w_{2}+\varepsilon }\right),\;P_{2}^{td}=\mathrm{Conv}\left(\frac{w_{1}^{\prime }\cdot C_{2}+w_{2}^{\prime }\cdot \mathrm{Upsample}\left(P_{3}^{td}\right)}{w_{1}^{\prime }+w_{2}^{\prime }+\varepsilon }\right),$$

where Upsample denotes bilinear interpolation, and the same-level skip connection $P_{i}^{skip}=C_{i}$ is preserved.

The bottom-up pathway subsequently refines the features with fine-grained spatial details:

(12) $$P_{2}^{out}=\mathrm{Conv}\left(\frac{w_{1}^{\prime \prime }\cdot C_{2}+w_{2}^{\prime \prime }\cdot P_{2}^{td}}{w_{1}^{\prime \prime }+w_{2}^{\prime \prime }+\varepsilon }\right),\;P_{3}^{out}=\mathrm{Conv}\left(\frac{w_{1}^{\prime \prime \prime }\cdot P_{3}^{td}+w_{2}^{\prime \prime \prime }\cdot \mathrm{Downsample}\left(P_{2}^{out}\right)+w_{3}^{\prime \prime \prime }\cdot C_{3}}{w_{1}^{\prime \prime \prime }+w_{2}^{\prime \prime \prime }+w_{3}^{\prime \prime \prime }+\varepsilon }\right),$$

(13) $$P_{4}^{out}=\mathrm{Conv}\left(\frac{w_{1}^{⁗}\cdot P_{4}^{td}+w_{2}^{⁗}\cdot \mathrm{Downsample}\left(P_{3}^{out}\right)+w_{3}^{⁗}\cdot C_{4}}{w_{1}^{⁗}+w_{2}^{⁗}+w_{3}^{⁗}+\varepsilon }\right),$$

where Downsample denotes strided convolution, and all *w* are learnable weights with w≥0 enforced by ReLU activation. The final outputs $\left\{P_{2}^{out},P_{3}^{out},P_{4}^{out}\right\}$ are passed to the detection head, with the notation clarified as $P_{i}^{out}=R_{i}$ to eliminate ambiguity.

#### 3.4.2. Morphology-Adaptive CIoU Loss

At the regression supervision level, we propose a morphology-adaptive CIoU (Complete IoU) loss that introduces a geometry-aware modulation factor, dynamically adjusting the strength of aspect ratio constraints according to the intrinsic shape of each target.

The standard CIoU loss is defined as:

(14) $$L_{\mathrm{CIoU}}=1-\mathrm{IoU}+\frac{\rho^{2}\left(b,b^{gt}\right)}{c^{2}}+\alpha v,$$

where:

(15) $$\mathrm{IoU}=\frac{|B\cap B^{gt}|}{|B\cup B^{gt}|},\;\rho^{2}\left(b,b^{gt}\right)={∥b-b^{gt}∥}_{2}^{2},$$

with *c* representing the diagonal length of the smallest enclosing box. The aspect ratio consistency term is:

(16) $$v=\frac{4}{\pi^{2}}{\left(\arctan\frac{w^{gt}}{h^{gt}}-\arctan\frac{w}{h}\right)}^{2},$$

and the balance coefficient is:

(17) $$\alpha =\frac{v}{(1-\mathrm{IoU})+v}.$$

However, in the standard formulation, α is determined solely by IoU and *v*, without accounting for target morphology. This is problematic for aerospace corrosion detection, where targets exhibit substantially different geometric characteristics. To address this limitation, we introduce a morphology-aware modulation factor β that adapts the aspect ratio constraint intensity based on the target’s aspect ratio *r*:

(18) $$r=\frac{\max(w^{gt},h^{gt})}{\min(w^{gt},h^{gt})},$$

defined as:

(19) $$\beta =\left\{\begin{array}{cc} 0.8, & r\leq 2\;(\mathrm{compact}\;\mathrm{morphology},\;\mathrm{characteristic}\;\mathrm{of}\;\mathrm{pitting}),\\ 1.0, & 2<r\leq 4\;(\mathrm{moderate}\;\mathrm{morphology},\;\mathrm{characteristic}\;\mathrm{of}\;\mathrm{diffuse}\;\mathrm{corrosion}),\\ 1.2, & r>4\;(\mathrm{elongated}\;\mathrm{morphology},\;\mathrm{characteristic}\;\mathrm{of}\;\mathrm{cracks}). \end{array}\right.$$

The morphology-adaptive balance coefficient becomes:

(20) $$\alpha_{\mathrm{corr}}=\beta \cdot \frac{v}{(1-\mathrm{IoU})+v},$$

yielding the final loss formulation:

(21) $$L_{\mathrm{Corr}\text{-}\mathrm{CIoU}}=1-\mathrm{IoU}+\frac{\rho^{2}\left(b,b^{gt}\right)}{c^{2}}+\beta \cdot \frac{v^{2}}{(1-\mathrm{IoU})+v}.$$

The threshold selection for β is grounded in the actual aspect-ratio distribution of the three corrosion morphologies in the AC-3H dataset. Pitting pits exhibit aspect ratios predominantly in the range 1≤r≤2, reflecting their approximately circular geometry. Diffuse corrosion regions typically fall within 2<r≤4 due to their irregular but moderately elongated shapes. Cracks, in contrast, consistently demonstrate aspect ratios r>4, often exceeding 10:1, reflecting their thread-like propagation along weld lines or stress directions. These empirically observed distributions naturally motivate the three-tier partitioning. The specific values β=0.8,1.0,1.2 were then selected through grid search on the validation set, confirming that intensified constraints improve crack localization while relaxed constraints benefit pitting detection. Thus, the thresholds are not arbitrary but directly correspond to the physical geometry of each corrosion type.

This adaptive strategy ensures that: for elongated cracks (r>4), aspect ratio constraints are intensified (β=1.2) to preserve thread-like continuity and prevent premature truncation; for compact pitting (r≤2), constraints are relaxed (β=0.8) to avoid over-regularization that might shift optimization focus away from center alignment; for diffuse corrosion (2<r≤4), balanced constraints (β=1.0) maintain standard optimization while accommodating inherent spatial uncertainty. This paradigm ensures that the regression objective remains consistently aligned with the intrinsic geometry of each corrosion type, fundamentally enhancing localization precision for irregularly shaped targets that would otherwise be poorly fitted by fixed-shape prior assumptions. The complete geometric calibration pipeline is summarized in Algorithm 3.
**Algorithm 3** Dual-Pronged Geometric Calibration  1:**Require:** Backbone features $\left\{C_{2},C_{3},C_{4}\right\}$, learnable fusion weights *w*  2:**Ensure:** Enhanced features $\left\{P_{2},P_{3},P_{4}\right\}$ and morphology-adaptive loss $L_{\mathrm{Corr}-\mathrm{CIoU}}$  3:**Stage 1: Customized BiFPN Fusion**  4:    Initialize $w_{2},w_{3},w_{4}\leftarrow 1.0$  5:    Top-down path:  6:       $P_{4}\leftarrow \mathrm{Conv}\left(C_{4}\right)$  7:       $P_{3}\leftarrow \mathrm{Resize}\left(P_{4}\right)+C_{3}$  8:       $P_{2}\leftarrow \mathrm{Resize}\left(P_{3}\right)+C_{2}$  9:    Weighted fusion:10:       $P_{i}=\mathrm{Conv}\left(\frac{\sum_{j}w_{j}\cdot \mathrm{Resize}\left(F_{j}\right)}{\varepsilon +\sum_{j}w_{j}}\right)$11:    Bottom-up path:12:       $R_{2}\leftarrow \mathrm{Conv}\left(P_{2}\right)$13:       $R_{3}\leftarrow \mathrm{Resize}\left(R_{2}\right)+P_{3}$14:       $R_{4}\leftarrow \mathrm{Resize}\left(R_{3}\right)+P_{4}$15:**Stage 2: Morphology-Adaptive Regression**16:    Compute IoU, $\rho^{2}$, *v* from predictions *B* and ground-truth $B^{gt}$17:    Compute aspect ratio: $r=\max\left(w^{gt},h^{gt}\right)/\min\left(w^{gt},h^{gt}\right)$18:    Determine morphology factor:19:       $\beta =\left\{\begin{array}{cc} 0.8, & r\leq 2\;(\mathrm{compact}\;\mathrm{pitting})\\ 1.0, & 2<r\leq 4\;(\mathrm{diffuse}\;\mathrm{corrosion})\\ 1.2, & r>4\;(\mathrm{elongated}\;\mathrm{cracks}) \end{array}\right.$20:    Compute morphology-adaptive CIoU loss:21:       $L_{\mathrm{Corr}-\mathrm{CIoU}}=1-\mathrm{IoU}+\frac{\rho^{2}}{c^{2}}+\beta \cdot \frac{v^{2}}{(1-\mathrm{IoU})+v}$22:**Return:** $\left\{P_{2},P_{3},P_{4}\right\}$, $L_{\mathrm{Corr}-\mathrm{CIoU}}$

The three modules of CorrSense are not independent components but form a synergistic cascade precisely engineered to address the intertwined challenges of aerospace corrosion detection. The dual-path data ecosystem directly tackles data scarcity by expanding the training distribution with physically plausible synthetic samples, ensuring that rare corrosion morphologies and extreme cases are represented during training. The saliency-based feature enhancement with SimAM addresses micro-target perceptual saliency by selectively amplifying responses at statistical outliers, precisely where micro-corrosion resides, while suppressing uniform background activations with zero parameter overhead, ensuring that even the weakest corrosion signals survive through the feature extraction pipeline. The dual-pronged geometric calibration strategy conquers the localization bottleneck: the customized BiFPN dynamically orchestrates cross-scale features according to target scale and morphology, ensuring that fine-grained spatial details from shallow layers are preserved alongside semantic discriminability from deeper layers; the morphology-adaptive CIoU loss ensures that regression supervision remains consistently aligned with the intrinsic geometry of each corrosion type, from compact pitting to thread-like cracks to ambiguous diffuse corrosion. Together, these innovations form a coherent framework where each component addresses a specific bottleneck while synergistically reinforcing the others, enabling robust and precise corrosion detection in the challenging aerospace domain.

## 4. Experiment Results

### 4.1. Dataset

To evaluate the effectiveness of the proposed CorrSense framework, we conduct experiments on the AC-3H (Aerospace Corrosion-3H) dataset. AC-3H is the first dedicated corrosion detection dataset for aerospace ground facilities under “three-high” marine environments (high temperature, high humidity, and high salt spray).

All real corrosion images in this study were collected from the Wenchang Space Launch Site in Hainan, China. The launch site is located at 19 degrees north latitude, adjacent to the South China Sea, and features a typical tropical maritime climate. According to meteorological statistics from the launch site, the annual average relative humidity reaches 86%, occasionally approaching 100% in certain months. The atmospheric Cl^−^ concentration in the Wenchang area ranges from 0.056 to 0.225 mg/m^3^, which ranks among the highest in China, with HCl concentrations ranging from 0.008 to 0.077 mg/m^3^. During midday, the surface temperature of steel structures can peak above 65 °C. The combined effects of prolonged high-temperature radiation, marine salt deposition, and high-humidity condensation not only accelerate electrochemical corrosion processes but also introduce significant imaging interferences: surface moisture on metals intensifies specular reflections, salt spray particle adhesion produces high-frequency noise, and coating degradation results in extensive color shifts and texture roughness variations. These factors substantially reduce the separability between corrosion regions and backgrounds compared to inland industrial environments, directly increasing the dependency of detection performance on data quality.

The acquisition targets cover the main steel structures of the launch tower, umbilical arm components, outer walls of cryogenic fuel storage tanks, ground gas pipelines, and various service facilities. The materials include Q345B structural steel, 5A06 aluminum alloy, and multiple aerospace-specific thermal control and protective coatings. To minimize the impact of varying capture conditions on data distribution, the acquisition process adhered to the principles of multi-angle coverage, prioritization of close-range details, and avoidance of strong reflections. For each corrosion region, multiple samples were captured from frontal, oblique, and varying distances to cover appearance variations under perspective changes. For metal surfaces prone to glare, acquisition was scheduled during periods with smaller illumination incident angles, or the shooting angle was adjusted to prevent texture loss due to overexposure. After preliminary on-site screening and post-processing elimination, 420 raw images were obtained. Secondary filtering was performed based on the following criteria: (1) removal of images with severe defocus causing detail loss, (2) removal of images with overexposed regions due to strong reflections that could not be recovered, and (3) removal of negative samples without any corrosion instances. This process yielded 300 valid corrosion images, which were uniformly resized to 640 × 640 pixels to balance detail preservation with storage overhead and training efficiency. Table 1 summarizes the acquisition conditions.

Regarding image preprocessing, we adopted resizing rather than cropping to unify all images to 640 × 640 pixels, based on the following considerations. First, cropping may discard corrosion targets located at image edges, particularly elongated cracks that often extend to frame boundaries, where cropping would cause critical information loss. Second, resizing preserves the complete structural context, facilitating the model’s learning of associations between corrosion and surrounding elements such as weld seams and bolts. Although resizing slightly compresses the pixel footprint of small targets, this issue is effectively mitigated through subsequent multi-scale training and data augmentation strategies. Therefore, resizing represents a reasonable choice balancing information integrity and computational efficiency.

The corrosion defects in the 300 images are classified into three typical morphologies based on on-site inspection records: pitting, cracks, and diffuse corrosion. Pitting appears as discretely distributed dark pits on metal surfaces, with diameters typically ranging from 0.5 mm to 2 mm, occupying only 20 to 60 pixels in 640 × 640 resolution images. Cracks typically develop along weld heat-affected zones or bolt hole edges, featuring extremely narrow widths with lengths spanning from several millimeters to tens of centimeters, generally exhibiting aspect ratios exceeding 10:1. Diffuse corrosion presents as extensive coating peeling or uniform substrate erosion, characterized by non-closed boundaries and gradual color transitions. The three defect types exhibit significant differences in boundary clarity: pitting and cracks have relatively well-defined visible boundaries, while diffuse corrosion often presents wide transition zones, imposing higher demands on bounding box annotation consistency. This classification primarily serves to understand defect appearances and annotation boundary variations. At the detection output level, to reduce category imbalance and fine-grained discrimination difficulty, we unify the three types into a single “corrosion” category, predicting only the bounding box positions. This approach focuses the study on achieving high-recall localization under complex backgrounds while providing a unified target for subsequent controllable augmentation.

Annotation was performed using LabelMe with standardized guidelines to minimize subjective variations. For pitting and cracks, bounding boxes were required to tightly fit the visible defect regions with a maximum margin of 5 pixels on each side, avoiding the inclusion of excessive background textures for the sake of regularity. For diffuse corrosion, boundaries were determined based on color mutation and texture transition locations; when transition zones were excessively wide, the median line of the transition zone was used as the box boundary, and these samples were marked as boundary-ambiguous for subsequent analysis. To further ensure annotation quality, all annotations were reviewed by an expert with higher experience after the initial labeling, focusing on box offset, scale deviation, and missed instances. For highly debatable weak candidates, instances with annotation box areas smaller than 16 × 16 pixels were excluded to prevent introducing uncertainty into the training data. Through this process, 1847 valid corrosion instances were generated from the 300 real images.

The scale distribution of the 1847 annotated instances is presented in Table 2. Small targets (area ≤ 32 × 32 pixels) account for 41.3% of all instances, highlighting the predominance of micro-corrosion targets in this dataset.

In addition to the real images, the AC-3H dataset incorporates high-fidelity synthetic samples generated through the diffusion-driven augmentation pipeline described in Section 3.2. The dataset adopts a scene-level partitioning strategy, dividing 120 independent scenes into training, validation, and test sets at an 8:1:1 ratio. The training set comprises 240 real images and 560 synthetic images, while the validation and test sets each contain 30 real images. All experimental results reported in this section are evaluated on the test set to ensure unbiased assessment of model generalization.

**Figure 3:**
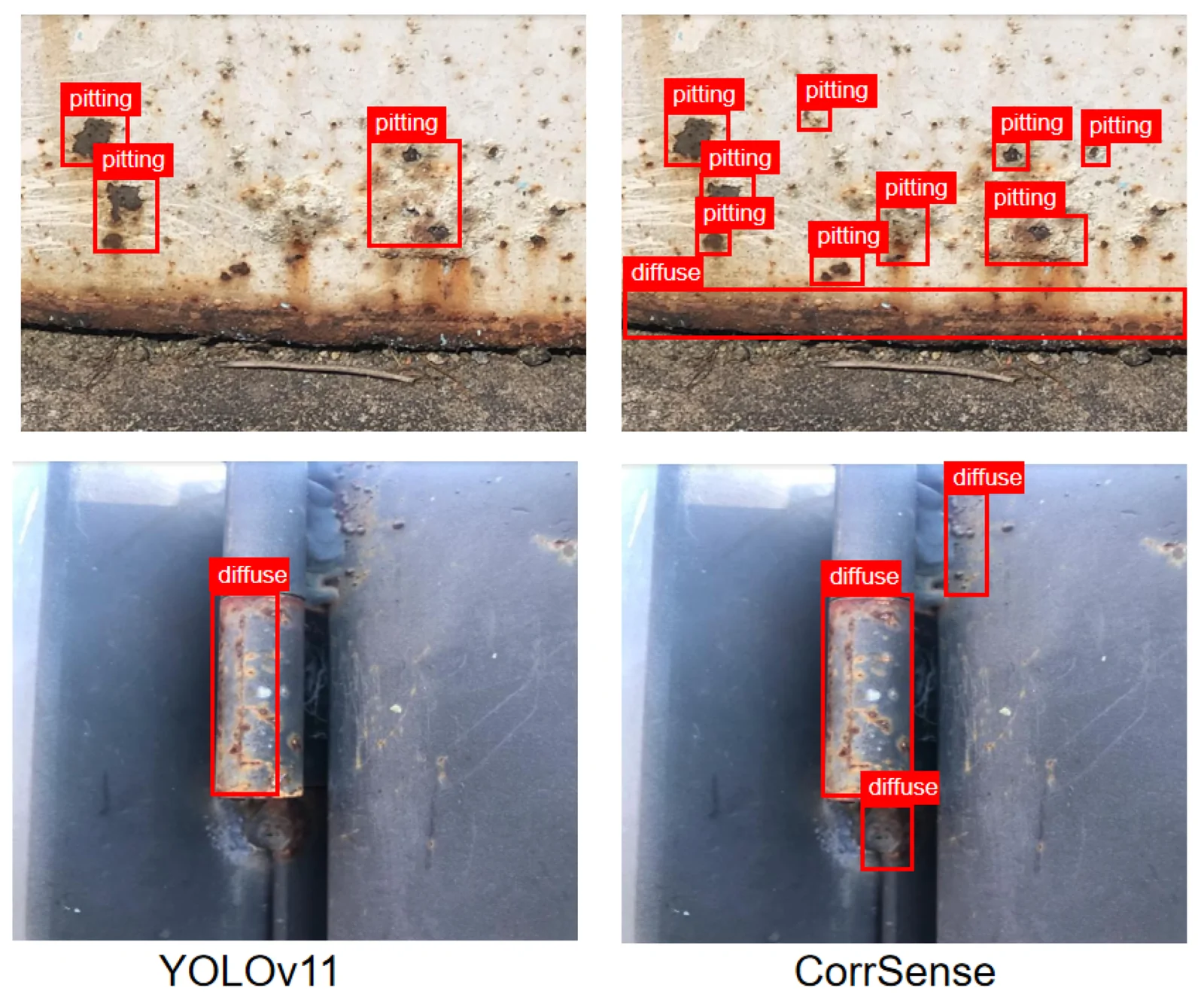
Detection results of CorrSense and YOLOv11 on representative challenging samples.

**Clarification on Morphology Labels and Task Definition.** Although three corrosion morphologies (pitting, cracks, and diffuse corrosion) are distinguished during the annotation stage to facilitate understanding of defect appearances, we emphasize that all morphology labels are unified into a single “corrosion” category for training and evaluation. Specifically, the detection head outputs only bounding boxes without morphology classification. The per-morphology IoU analysis presented is a post-hoc evaluation conducted solely to analyze the model’s localization performance across different geometric characteristics; these morphology labels are not used as training supervision. In Figure 3, the labels “pitting” and “diffuse” are shown purely for visualization purposes to indicate the ground-truth morphology type of each instance, not to imply that the model performs morphology classification. This clarification ensures the task definition remains consistent as a single-class detection problem.

**Dataset Representativeness Analysis.** To quantitatively justify the diversity and representativeness of the AC-3H dataset despite its moderate size, we provide a detailed statistical characterization. As summarized in Table 3, the 300 real images capture three distinct corrosion morphologies across diverse structural contexts, with substantial variation in target scales and background conditions. The dataset covers 120 independent scenes, ensuring that the 30 test images are drawn from scenes completely unseen during training. The 41.3% proportion of small targets (≤32 × 32 pixels) reflects the practical challenge of micro-corrosion detection. While we acknowledge the dataset size as a current limitation, the carefully curated scene diversity and the challenging distribution of target scales provide a meaningful and realistic benchmark for aerospace corrosion detection.

**Data Leakage Prevention.** To prevent potential data leakage, we emphasize that all synthetic samples are generated exclusively from the intact images corresponding to the **training scenes**. The validation and test scenes are strictly isolated throughout the entire generation process, no images from the validation or test scenes, whether intact or corroded, are used for diffusion model fine-tuning, DDIM inversion, or synthetic sample generation. This strict scene-level separation guarantees that the evaluation results on the test set reflect genuine generalization performance rather than memorization of scene-specific patterns.

### 4.2. Baseline Methods and Implementation Details

We compare the proposed CorrSense against several state-of-the-art object detection methods spanning both CNN-based and transformer-based architectures to comprehensively demonstrate its effectiveness. The baseline methods include:

- **Faster R-CNN**: A classic two-stage detector that employs a Region Proposal Network (RPN) to generate candidate regions followed by a separate detection head for classification and bounding box regression. It remains a strong representative of two-stage detection paradigms.
- **DETR**: An end-to-end transformer-based detector that eliminates the need for hand-crafted components such as NMS and anchor generation by formulating object detection as a direct set prediction problem.
- **RT-DETR-L**: A real-time transformer-based detector that introduces a hybrid encoder and an IoU-aware query selection mechanism, achieving efficient and accurate detection without NMS post-processing.
- **YOLOv8**: A widely adopted version of the YOLO family featuring an anchor-free design and enhanced feature extraction capabilities.
- **YOLOv9**: An improved YOLO variant incorporating Programmable Gradient Information (PGI) and Generalized Efficient Layer Aggregation Network (GELAN).
- **YOLOv11**: The baseline backbone of our framework, representing the latest advancement in the YOLO series with optimized architecture and training strategies.

For fair comparison, all methods are trained and evaluated under identical conditions: an input resolution of 640 × 640 pixels, the SGD optimizer with an initial learning rate of 0.01, momentum of 0.937, weight decay of 0.0005, and 200 training epochs. For transformer-based methods (DETR and RT-DETR-L), we adopt their default optimizer settings (AdamW with initial learning rate of 1 × $10^{-4}$) while keeping all other training configurations consistent. All experiments are conducted on an NVIDIA Tesla V100 GPU with FP16 mixed precision training. The proposed CorrSense framework shares the same training configuration, with the three proposed modules (diffusion-driven augmentation, SimAM enhancement, and customized BiFPN with morphology-adaptive CIoU) incrementally integrated to isolate their individual contributions.

### 4.3. Evaluation Metrics

To comprehensively evaluate detection performance and localization accuracy, we adopt the following metrics:

- **mAP@0.5**: Mean Average Precision at IoU threshold 0.5, reflecting the overall detection capability for corrosion targets.
- **mAP@0.5:0.95**: Mean Average Precision averaged over IoU thresholds from 0.5 to 0.95 (step size 0.05), providing a more stringent assessment of localization precision.
- **Recall_small_**, **Recall_medium_**, **Recall_large_**: Per-scale recall rates for targets with areas smaller than $32^{2}$ pixels, between $32^{2}$ and $96^{2}$ pixels, and larger than $96^{2}$ pixels, respectively.
- **Precision** and **Recall**: Standard precision and recall metrics for overall detection performance.
- **Mean IoU**: The average Intersection over Union between predicted and ground-truth bounding boxes, directly reflecting localization quality.
- **Per-morphology IoU**: IoU statistics computed separately for pitting, cracks, and diffuse corrosion to reveal the method’s effectiveness on different corrosion types.

### 4.4. Implementation Details of Diffusion Pipeline

To ensure the reproducibility of our diffusion-driven augmentation pipeline, we provide the complete set of hyperparameters and implementation details. The pre-trained Stable Diffusion v1.5 is adopted as the base generative model. For domain adaptation, we employ LoRA with rank r=8, targeting the query, key, and value projection matrices in the cross-attention layers. The LoRA fine-tuning is performed with a learning rate of $1\times 10^{-4}$ for 5000 training steps on the 300 real corrosion images.

For controllable generation, we use DDIM inversion with N=50 steps and a guidance scale of 7.5. Example textual prompts include: “pitting corrosion on aerospace steel surface under salt spray environment” and “stress corrosion cracking along weld seam on launch tower structure.” The Frechet Inception Distance threshold is set to τ=15, and the actual achieved FID on the retained synthetic set is 13.7. From 2200 initial candidates, we retain 560 high-fidelity synthetic images.

For the hybrid annotation verification of synthetic samples, two domain experts with over three years of experience in aerospace corrosion inspection perform the verification using LabelMe, following the same annotation protocol described in Section 3.1. Inter-annotator agreement is assessed on a random 20% subset, achieving an IoU consistency exceeding 0.9. All parameters are summarized in Table 4.

### 4.5. Research Questions

To systematically validate the proposed framework, we formulate the following research questions:

- **RQ1: Data Ecosystem Effectiveness.** Does the dual-path data ecosystem with diffusion-driven augmentation effectively address the data scarcity challenge? Does the augmented dataset lead to consistent improvements in detection performance compared to training solely on real data?
- **RQ2: SimAM Feature Enhancement.** Does the saliency-based feature enhancement strategy with parameter-free SimAM improve the perceptual saliency of micro-corrosion targets? How does it affect small-target recall and overall detection accuracy?
- **RQ3: Geometric Calibration.** Does the dual-pronged geometric calibration strategy with customized BiFPN and morphology-adaptive CIoU enhance localization precision, particularly for irregularly shaped corrosion targets such as elongated cracks and diffuse corrosion?
- **RQ4: Overall Framework Efficacy.** Does the complete CorrSense framework, integrating all three innovations, achieve superior performance compared to state-of-the-art detection methods on challenging aerospace corrosion samples?

### 4.6. Experimental Results

#### 4.6.1. RQ1: Data Ecosystem Effectiveness

To assess the effectiveness of the dual-path data ecosystem with diffusion-driven augmentation, we conduct ablation experiments comparing models trained with and without augmented data. Specifically, we train the baseline YOLOv11 under two configurations: (1) real data only (300 real images), and (2) real data combined with high-fidelity synthetic samples generated by our diffusion-driven augmentation pipeline (300 real + 560 synthetic images). All other training configurations remain identical. Table 5 presents the comprehensive results across all baseline methods and evaluation metrics.

The results demonstrate that the diffusion-driven augmentation yields substantial and consistent improvements across all methods and metrics. The most pronounced gains are observed in small-target recall, with the YOLOv11 baseline achieving a remarkable 15.7% absolute improvement from 0.718 to 0.875. This confirms that the generated synthetic samples effectively enrich the training distribution with rare micro-corrosion patterns. The consistent improvements across all methods, ranging from 6.1% to 15.7% in Recall_small_, validate that the augmented data not only helps detect small targets but also enhances overall detection robustness. Notably, the improvement is more significant for methods with lower baseline performance, suggesting that data augmentation is particularly beneficial for models that struggle with limited training data.

**Control Experiments with Matched Dataset Size.** To isolate the effect of diffusion-driven synthesis from the increase in training set size, we conduct additional experiments with matched total sample sizes (860 images). Using YOLOv11 as the representative detector, we compare our method against four alternative augmentation strategies at identical data scales. Classical Augmentation applies random rotation, flipping, scaling, and color jitter to existing samples, yet fails to introduce new semantic variations. Copy-Paste extracts and pastes corrosion instances onto intact backgrounds but may introduce illumination or scale inconsistencies. GAN-based Augmentation employs a StyleGAN trained on real corrosion data, though it suffers from training instability and mode collapse under limited data. Full SD Fine-tuning (non-LoRA) fully tunes the pre-trained Stable Diffusion model on the 300 real images, but is prone to overfitting due to the large parameter space relative to the limited sample size. As shown in Table 6, our LoRA + DDIM approach consistently outperforms all alternatives.

**Synthetic-to-Real Ratio Sweep.** To justify the selection of 560 synthetic samples, we conduct a systematic sweep over synthetic-to-real ratios using YOLOv11. As shown in Table 7, performance saturates at approximately 560 synthetic samples (1.87:1 ratio), beyond which improvements are marginal. We therefore select this ratio as the optimal trade-off between performance gain and computational efficiency.

#### 4.6.2. RQ2: SimAM Feature Enhancement Effectiveness

To evaluate the contribution of the SimAM-based saliency-based feature enhancement strategy, we compare the performance of YOLOv11 baseline with and without the SimAM module inserted in the shallow C2 and C3 layers. All other configurations remain identical. Table 8 presents the comprehensive comparison across all baseline methods, where each method is evaluated with and without the SimAM enhancement applied to its backbone.

The SimAM module yields the most significant improvement in small-target recall across all methods, with the YOLOv11 baseline achieving a 4.1% absolute increase from 0.875 to 0.916. This confirms that the saliency-based feature enhancement selectively amplifies neuronal responses at micro-corrosion locations while suppressing background activations, effectively alleviating the low signal-to-noise ratio problem in shallow feature maps. Notably, this gain is achieved without introducing any learnable parameters, preserving the model’s computational efficiency. The improvement is consistently more pronounced for small targets than for medium or large targets across all methods, validating the targeted design of SimAM for addressing micro-target perceptual saliency. Furthermore, the precision remains stable or slightly improves, indicating that the enhanced recall does not come at the cost of increased false positives.

#### 4.6.3. RQ3: Geometric Calibration Effectiveness

To validate the dual-pronged geometric calibration strategy, we conduct ablation experiments evaluating the individual and combined contributions of customized BiFPN and morphology-adaptive CIoU. All models in this experiment are built upon the SimAM-enhanced baseline. Table 9 presents the comprehensive results across all methods, where each method is evaluated with different geometric calibration configurations.

The customized BiFPN contributes a 1.6% improvement in mAP@0.5:0.95 and increases mean IoU from 0.828 to 0.840, demonstrating that dynamic cross-scale feature orchestration enhances multi-scale target localization. The morphology-adaptive CIoU further improves mAP@0.5:0.95 by 1.2% and elevates mean IoU to 0.851. Most notably, the IoU for crack targets shows the most substantial improvement, increasing by 3.7% from the baseline to 0.822. This confirms that the adaptive aspect ratio constraint is particularly effective for elongated and irregularly shaped corrosion targets. The combined effect of both components yields the best overall performance, validating the dual-pronged geometric calibration design. Across all methods, the geometric calibration consistently improves IoU metrics, with larger gains observed for crack targets, followed by diffuse corrosion and pitting. This gradient is consistent with the design rationale: elongated cracks benefit most from adaptive aspect ratio constraints, while compact pitting benefits less but still achieves meaningful improvement.

#### 4.6.4. RQ4: Overall Framework Efficacy

To evaluate the overall effectiveness of the complete CorrSense framework integrating all three innovations, we compare it against all baseline methods on the AC-3H test set. Table 10 presents the comprehensive performance comparison.

CorrSense consistently outperforms all baseline methods across every evaluation metric. Compared to the baseline YOLOv11, our framework achieves a 2.2% absolute improvement in mAP@0.5 and a substantial 3.8% improvement in mAP@0.5:0.95, demonstrating that the proposed enhancements significantly boost both detection capability and localization precision. Notably, the improvement is most pronounced for small targets, where CorrSense outperforms the baseline by 4.6% in Recall_small_, confirming the effectiveness of the SimAM-based saliency-based feature enhancement strategy. The consistent gains across all scales and metrics validate that the three synergistic innovations collectively address the intertwined challenges of data scarcity, micro-target saliency, and irregular morphology localization. Compared to the strongest competitor RT-DETR-L, CorrSense achieves a 0.9% improvement in mAP@0.5 and a more substantial 3.6% improvement in mAP@0.5:0.95, highlighting the particular advantage of our framework in achieving high-precision localization.

Figure 3 visualizes the detection performance of YOLOv11 and CorrSense on pitting and diffuse corrosion samples. CorrSense consistently delivers more comprehensive and fine-grained detections, successfully capturing subtle pitting pits missed by YOLOv11 and producing tighter, more accurate bounding boxes for diffuse corrosion regions with ambiguous boundaries. These results qualitatively confirm that CorrSense achieves superior sensitivity and localization precision for challenging corrosion targets.

## 5. Conclusions

In this paper, we have presented CorrSense, a YOLOv11-based intelligent corrosion sensing and detection framework specifically designed for aerospace ground facilities operating under harsh “three-high” marine environments. To overcome the critical challenge of data scarcity, we constructed AC-3H, the first dedicated corrosion dataset for aerospace facilities, and developed a diffusion-driven augmentation engine that synthesizes physically plausible corrosion samples with structural fidelity preserved. To address the low signal-to-noise ratio of micro-corrosion targets in shallow feature maps, we embedded a parameter-free SimAM attention mechanism into the shallow backbone layers, selectively amplifying responses at micro-corrosion locations while suppressing background activations without introducing learnable parameters. To tackle the localization difficulties caused by geometric irregularities, we formulated a dual-pronged geometric calibration strategy featuring a customized BiFPN with learnable weighted fusion and a morphology-adaptive CIoU loss that modulates aspect ratio constraints according to target shape. Extensive experiments on the AC-3H test set demonstrate that CorrSense consistently outperforms state-of-the-art detectors including Faster R-CNN, DETR, RT-DETR-L, YOLOv8, YOLOv9, and the baseline YOLOv11 across all evaluation metrics, achieving 94.5% mAP@0.5 and 62.5% mAP@0.5:0.95, with particularly substantial gains in small-target recall and localization precision for irregularly shaped corrosion targets. These results validate that the proposed framework effectively addresses the intertwined challenges of data scarcity, micro-target saliency, and irregular morphology localization, providing a promising solution for intelligent corrosion inspection in coastal launch sites.

Despite the promising results, we acknowledge several limitations that should be addressed in future work. First, while the AC-3H dataset provides a valuable benchmark for aerospace corrosion detection, it remains limited in scale, comprising 300 real images collected from a single launch site. The current evaluation, though rigorous within this scope, does not fully establish the generalizability of CorrSense across different launch sites, climatic conditions, or facility types. To address this, we plan to expand the dataset through continued data collection at multiple coastal launch sites and to conduct cross-site validation studies to systematically assess the framework’s robustness to domain shifts. Second, the current framework is evaluated exclusively on bounding-box-level detection; extending it to pixel-level segmentation would enable more precise corrosion severity assessment, which we leave as future work.

For future work, we plan to extend the framework in several directions. First, building upon the insights from FEK-AML and other few-shot learning methods, we will explore meta-learning and active learning strategies to enable the model to rapidly adapt to new corrosion patterns, unseen facility types, and varying launch site conditions with minimal human supervision. Second, we aim to incorporate physics-guided priors and knowledge graph-based reasoning into the detection pipeline, enabling not only defect localization but also causal reasoning about corrosion evolution, severity assessment, and maintenance decision support—moving toward trustworthy multi-step prediction for structural health monitoring. Third, we intend to extend the current detection paradigm to instance segmentation for pixel-level corrosion delineation, facilitating more accurate corrosion severity assessment and remaining-life prediction. Fourth, we will investigate model compression and hardware acceleration techniques to enable real-time deployment on edge devices for practical on-site inspection applications.

## Figures and Tables

**Table 1:** Acquisition setup of real corrosion images.

Item	Description
Acquisition Location	Wenchang Space Launch Site, Hainan, China
Environmental Characteristics	Typical tropical maritime climate: high temperature, high humidity, high salt spray
Annual Average Relative Humidity	86%
Cl^−^ Concentration	0.056–0.225 mg/m^3^
Peak Surface Temperature	Steel structures exceed 65 °C at midday
Acquisition Targets	Launch tower steel structures, umbilical arms, cryogenic tank outer walls, ground pipelines, service facilities
Primary Materials	Q345B structural steel, 5A06 aluminum alloy, various protective coatings
Raw Captured Images	420
Valid Images After Screening	300
Unified Resolution	640 × 640
Screening Criteria	Removal of defocused images, overexposed images, and images without corrosion instances

**Table 2:** Scale distribution of real corrosion samples.

Scale Level	Instance Count	Proportion (%)
Small (≤32 × 32)	763	41.3
Medium (32×32<area≤96×96)	622	33.7
Large (>96 × 96)	462	25.0
Total	1847	100.0

**Table 3:** Diversity and representativeness statistics of the AC-3H dataset.

Diversity Dimension	Value	Significance
Total Real Images	300	Real-world operational data
Independent Scenes	120	Scene-level test isolation
Corrosion Morphologies	3 (pitting, cracks, diffuse)	Morphological diversity
Small Target Proportion	41.3%	Micro-target detection challenge
Backgrounds with Structural Overlap	61.0%	Complex background modeling
Annotation Instances	1847	Statistical reliability

**Table 4:** Hyperparameters of the diffusion-driven augmentation pipeline.

Parameter	Value
Base Model	Stable Diffusion v1.5
LoRA Rank	r=8
LoRA Target Modules	Q, K, V in cross-attention
LoRA Learning Rate	$1\times 10^{-4}$
Training Steps	5000
DDIM Inversion Steps	N=50
Guidance Scale	7.5
FID Threshold (τ)	15
Achieved FID	13.7
Candidates Generated	2200
Candidates Retained	560
Annotators	2 domain experts (3+ years)
Inter-Annotator Agreement	IoU > 0.9 on 20% subset

**Table 5:** RQ1: Effectiveness of diffusion-driven augmentation on the AC-3H test set. The results demonstrate consistent improvements brought by synthetic data generation.

Detector	Data	mAP@0.5	mAP@0.5:0.95	R_s_	R_m_	R_l_	Precision
Faster R-CNN	Real Only	0.798	0.462	0.701	0.758	0.801	0.851
	Real + Synth	0.862	0.501	0.762	0.812	0.854	0.878
DETR	Real Only	0.812	0.478	0.715	0.772	0.815	0.862
	Real + Synth	0.875	0.518	0.781	0.835	0.868	0.884
RT-DETR-L	Real Only	0.852	0.505	0.782	0.821	0.855	0.883
	Real + Synth	0.918	0.561	0.848	0.881	0.908	0.912
YOLOv8	Real Only	0.838	0.492	0.772	0.798	0.835	0.872
	Real + Synth	0.904	0.535	0.841	0.862	0.895	0.899
YOLOv9	Real Only	0.846	0.501	0.788	0.806	0.842	0.878
	Real + Synth	0.912	0.548	0.852	0.873	0.902	0.905
YOLOv11	Real Only	0.841	0.523	0.718	0.835	0.892	0.892
	Real + Synth	**0.923**	**0.587**	**0.875**	**0.901**	**0.918**	**0.911**
**Absolute Improvement (percentage points) (%)**							
Faster R-CNN	–	+6.4	+3.9	+6.1	+5.4	+5.3	+2.7
DETR	–	+6.3	+4.0	+6.6	+6.3	+5.3	+2.2
RT-DETR-L	–	+6.6	+5.6	+6.6	+6.0	+5.3	+2.9
YOLOv8	–	+6.6	+4.3	+6.9	+6.4	+6.0	+2.7
YOLOv9	–	+6.6	+4.7	+6.4	+6.7	+6.0	+2.7
YOLOv11	–	**+8.2**	**+6.4**	**+15.7**	+6.6	+2.6	+1.9

**Table 6:** Comparison of augmentation strategies at matched dataset size using YOLOv11 (860 training images).

Augmentation Strategy	mAP@0.5	mAP@0.5:0.95	R_s_	Precision
Classical Augmentation	0.885	0.552	0.828	0.897
Copy-Paste	0.901	0.568	0.851	0.904
GAN-based Augmentation	0.893	0.558	0.835	0.899
Full SD (non-LoRA)	0.912	0.579	0.862	0.908
**Ours (LoRA + DDIM)**	**0.923**	**0.587**	**0.875**	**0.911**

**Table 7:** Synthetic-to-real ratio ablation study using YOLOv11.

Ratio	Synthetic Count	mAP@0.5	mAP@0.5:0.95	R_s_	Training Cost
0.5:1	150	0.892	0.558	0.832	0.8×
1:1	300	0.908	0.573	0.851	1.0×
1.87:1	560	0.923	0.587	0.875	1.2×
3:1	900	0.925	0.589	0.878	1.6×
5:1	1500	0.926	0.590	0.879	2.3×

**Table 8:** RQ2: Effectiveness of SimAM shallow feature enhancement on the AC-3H test set. The results demonstrate that SimAM consistently improves feature representation and detection performance.

Detector	SimAM	mAP@0.5	mAP@0.5:0.95	R_s_	R_m_	R_l_	Precision
Faster R-CNN	Without	0.862	0.501	0.762	0.812	0.854	0.878
	With	0.872	0.509	0.788	0.818	0.858	0.883
DETR	Without	0.875	0.518	0.781	0.835	0.868	0.884
	With	0.883	0.525	0.805	0.841	0.872	0.889
RT-DETR-L	Without	0.918	0.561	0.848	0.881	0.908	0.912
	With	0.924	0.568	0.872	0.887	0.912	0.916
YOLOv8	Without	0.904	0.535	0.841	0.862	0.895	0.899
	With	0.911	0.542	0.869	0.868	0.899	0.903
YOLOv9	Without	0.912	0.548	0.852	0.873	0.902	0.905
	With	0.918	0.555	0.878	0.879	0.906	0.909
YOLOv11	Without	0.923	0.587	0.875	0.901	0.918	0.911
	With	**0.937**	**0.597**	**0.916**	0.901	0.918	**0.918**
**Absolute Improvement (percentage points) (%)**							
Faster R-CNN	–	+1.0	+0.8	+2.6	+0.6	+0.4	+0.5
DETR	–	+0.8	+0.7	+2.4	+0.6	+0.4	+0.5
RT-DETR-L	–	+0.6	+0.7	+2.4	+0.6	+0.4	+0.4
YOLOv8	–	+0.7	+0.7	+2.8	+0.6	+0.4	+0.4
YOLOv9	–	+0.6	+0.7	+2.6	+0.6	+0.4	+0.4
YOLOv11	–	**+1.4**	**+1.0**	**+4.1**	–	–	**+0.7**

**Table 9:** RQ3: Effectiveness of geometric calibration on the AC-3H test set. The combination of BiFPN and Morph-CIoU further improves localization accuracy and bounding box IoU quality.

Detector	Config	mAP@0.5	mAP@0.5:0.95	Mean IoU	Crack IoU	Pitting IoU	Diffuse IoU
Faster R-CNN	Baseline	0.872	0.509	0.748	0.712	0.735	0.778
	+ BiFPN	0.879	0.518	0.758	0.725	0.742	0.785
	+ Morph-CIoU	0.881	0.522	0.764	0.734	0.746	0.791
	+ Both	0.887	0.528	0.772	0.748	0.752	0.798
DETR	Baseline	0.883	0.525	0.765	0.728	0.752	0.795
	+ BiFPN	0.889	0.533	0.774	0.741	0.758	0.802
	+ Morph-CIoU	0.891	0.537	0.779	0.749	0.762	0.807
	+ Both	0.896	0.543	0.786	0.762	0.768	0.813
RT-DETR-L	Baseline	0.924	0.568	0.798	0.762	0.785	0.824
	+ BiFPN	0.929	0.578	0.808	0.776	0.792	0.831
	+ Morph-CIoU	0.931	0.582	0.814	0.785	0.796	0.836
	+ Both	0.936	0.589	0.822	0.798	0.802	0.842
YOLOv8	Baseline	0.911	0.542	0.778	0.742	0.765	0.805
	+ BiFPN	0.916	0.552	0.788	0.756	0.772	0.812
	+ Morph-CIoU	0.918	0.556	0.793	0.764	0.776	0.817
	+ Both	0.922	0.563	0.801	0.778	0.782	0.823
YOLOv9	Baseline	0.918	0.555	0.785	0.748	0.772	0.812
	+ BiFPN	0.923	0.564	0.795	0.762	0.778	0.819
	+ Morph-CIoU	0.925	0.568	0.801	0.772	0.783	0.824
	+ Both	0.929	0.575	0.808	0.785	0.788	0.831
YOLOv11	Baseline	0.937	0.597	0.828	0.785	0.812	0.852
	+ BiFPN	0.941	0.613	0.840	0.802	0.821	0.863
	+ Morph-CIoU	0.941	0.619	0.846	0.813	0.826	0.868
	**+ Both**	**0.945**	**0.625**	**0.851**	**0.822**	**0.831**	**0.874**
**Absolute Improvement (percentage points) (%)**							
YOLOv11	–	+0.8	+2.8	+2.3	+3.7	+1.9	+2.2

**Table 10:** RQ4: Overall performance comparison of CorrSense with state-of-the-art methods on the AC-3H test set. All competing methods are evaluated with their optimal configurations identified in the ablation studies (i.e., with BiFPN and Morph-CIoU applied where applicable). Best results are highlighted in **bold**.

Method	mAP@0.5	mAP@0.5:0.95	R_s_	R_m_	R_l_	Precision	Mean IoU
Faster R-CNN	0.887	0.528	0.798	0.834	0.868	0.891	0.772
DETR	0.896	0.543	0.815	0.849	0.879	0.895	0.786
RT-DETR-L	0.936	0.589	0.882	0.895	0.918	0.921	0.822
YOLOv8	0.922	0.563	0.886	0.892	0.907	0.914	0.801
YOLOv9	0.929	0.575	0.894	0.898	0.912	0.918	0.808
YOLOv11 (Baseline)	0.923	0.587	0.875	0.901	0.918	0.911	0.828
**CorrSense (Ours)**	**0.945**	**0.625**	**0.921**	**0.923**	**0.931**	**0.924**	**0.851**
**Absolute Improvement (percentage points) (%)**							
Improvement over YOLOv11	+2.2	+3.8	+4.6	+2.2	+1.3	+1.3	+2.3
Improvement over RT-DETR-L	+0.9	+3.6	+3.9	+2.8	+1.3	+0.3	+2.9

## Data Availability

The raw data supporting the conclusions of this article will be made available by the authors on request. Detailed training logs, hyperparameter configurations, and raw per-run results for all experiments are provided in the Supplementary Materials.

## Supplementary Materials

The following supporting information can be downloaded at: https://www.mdpi.com/article/10.3390/s26175504/s1.
